# Zwitterion Effect of Cow Brain Protein towards Efficiency Improvement of Dye-Sensitized Solar Cell (DSSC)

**DOI:** 10.1155/2020/7910702

**Published:** 2020-02-19

**Authors:** Denny Widhiyanuriyawan, Prihanto Trihutomo, Sudjito Soeparman, Lilis Yuliati

**Affiliations:** ^1^Department of Mechanical Engineering, Brawijaya University, Jl. Veteran, Malang 65145, Indonesia; ^2^Department of Mechanical Engineering, State University of Malang, Jl. Semarang 5, Malang 65145, Indonesia

## Abstract

Dye-Sensitized Solar Cell (DSSC) constitutes a solar cell using natural dyes from plants that are adsorbed in semiconductors to convert solar energy into electrical energy. DSSC has relatively inexpensive fabrication costs, is easy to produce, works in visible light, and is environmentally friendly. The disadvantage of DSSC is that its efficiency is still low compared to silicon solar cells. This low efficiency is due to obstacles in the flow of electric current on DSSC. In this study, DSSC has been successfully fabricated with the deposition of clathrin protein from cow brain. The zwitterions effect of protein on cow brain is able to reduce resistance and increase electric current on DSSC. The zwitterions effect of cow brain protein that fills gaps or empty spaces between TiO_2_ particles generates acidic reactions (capturing electrons) and bases (releasing electrons); hence, proteins in the cow brain are able to function as electron bridges between TiO_2_ molecules and generate an increase in electric current in DSSC. The method used in this research was to deposit clathrin protein from cow brain in a porous TiO_2_ semiconductor with a concentration of 0%, 25%, 50%, and 75%. Tests carried out on DSSC that have been performed were X-Ray Diffractometer (XRD) testing to determine the crystal structure formed, Fourier Transform Infrared Spectroscopy (FTIR) testing to determine the functional groups formed on DSSC, Scanning Electron Microscopy (SEM) testing to determine the surface morphological characteristics of the DSSC layer, and testing the efficiency using AM 1.5 G solar simulator (1000 W/m2) to determine the efficiency changes that occur in DSSC. From the XRD test results by increasing the concentration of cow brain protein in DSSC, the structure of amino acid crystals also increased and the crystal size increased with the largest crystal size of 42.25 nm at the addition of 75% of cow brain protein. FTIR test results show that the addition of cow brain protein will form functional protein-forming amino groups on DSSC. FTIR analysis shows the sharp absorption of energy by protein functional groups in the FTIR spectrum with increasing concentration of cow brain protein in DSSC. The SEM test results show that the concentration of additional molecules of protein deposited into TiO_2_ increases and the cavity or pore between the TiO_2_ molecules decreases. The reduction of cavities in the layers indicates that protein molecules fill cavities that exist between TiO_2_ molecules. From the results of testing using AM 1.5 G solar simulator (1000 W/m2), the highest efficiency value is 1.465% with the addition of 75% brain protein concentration.

## 1. Introduction

The technical obstacle to the development of solar cells is the need for a single crystal silicon to generate solar cell panels to produce an efficiency of around 30%. While producing single crystal silicon requires a high cost, hence it is not efficient as an alternative energy source. The low efficiency of solar cells causes the need for large amounts of solar panels to generate large amounts of power [1]. Another technical obstacle is the absorption spectrum that is too narrow from silicon-based solar cells. The main spectrum of absorption of silicon solar cells is ultraviolet and purple. It is known that the energy distribution from sunlight consists of about 7% of ultraviolet, 47% of visible light, and 46% of infrared. This shows that silicon solar cells cannot use almost 93% of the energy from sunlight [2].

To overcome this problem, a recent type of photochemical solar cell has been discovered, namely, Dye-Sensitized Solar Cell (DSSC), which is a type of solar cell exciton developed by O'Regan and Gratzel in 1991 [3].

**Figure 1:**
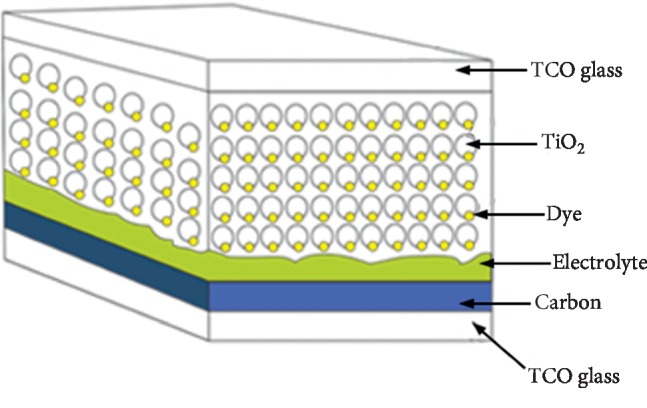
Dye-Sensitized Solar Cell structure [3].

Unlike conventional solar cells, DSSC is a photoelectrochemical solar cell that uses electrolytes as a charge transport medium. Besides electrolytes, DSSC is divided into several parts consisting of TiO_2_ nanopores, dye molecules adsorbed on the surface of TiO2, and carbon catalysts, which are all deposited between two Transparent Conductive Oxide (TCO) pieces of glass, as shown in Figure 1.

Some of the advantages offered by DSSC compared to other solar cell devices are, for instance, as follows: the fabrication costs are relatively inexpensive, it is easy to produce, it works in visible light, and it is environmentally friendly [4].

One of the most important DSSC components is a dye sensitizer, which is an electron pump driven by photos (light) from a DSSC device. This allows the injection of electrons into the semiconductor conduction band and the conversion of visible light photons into electricity. Dyes in DSSC act as absorbers of visible light or sensory photons; thus, the TiO_2_ semiconductor that only works in the UV region is able to work in the visible light region. Inorganic sensitizers in the form of synthetic transition metal complex dyes are reported to have good solar-to-electricity conversion efficiency [5]. Although it has been proven effective, the high cost of production (>$1000/g), limited ingredients, and side effects caused by the environment become obstacles when it will be implemented on a large scale [6].

Organic compounds from nature can also be used as a sensitizer in DSSC. Organic compounds as sensory dyes are less expensive, easier to obtain and produce, and environmentally friendly. Natural plant pigments such as chlorophyll, carotene, and anthocyanin extracted from leaves, flowers, fruit, and skin are reported to be used as sensitizers in DSSC [7]. The use of chlorophyll as a natural dye in DSSC has been carried out by Arifin et al. [8]. Wang et al. conducted a study using chlorophyllin isolated from *Undaria pinnatifida* seaweed for use as a sensitizer on DSSC [9].  Table 1 shows the comparison of efficiency in DSSC using a variety of different natural dyes.

According to Table 1, DSSC using a variety of natural dyes obtains efficiencies ranging 0.03%–1.7%.

**Figure 2:**
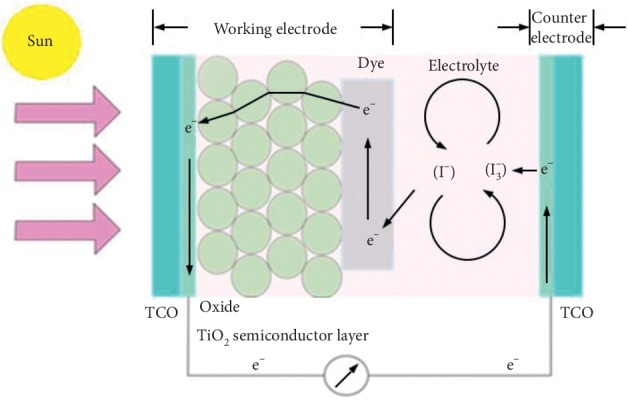
Working scheme of DSSC [3].

Basically, the working principle of DSSC is the reaction of electron transfer (Figure 2). The first process begins with the occurrence of electron excitation in dye molecules due to absorption of photons (hv). Electrons are excited from ground state (*D*) to excited state (*D*^*∗*^). The electrons from the excited state are then directly injected into the titania conduction band (ECB); hence, the dye molecule is oxidized (*D*^+^). After reaching the TCO electrode, electrons flow into the counter electrode through an external circuit. In the presence of a catalyst at the counter electrode, electrons are received by the electrolyte; accordingly, the holes formed in the electrolyte (I_3_^−^), due to electron donors in the previous process, combine with electrons to form iodide (I^−^). With the electron donor by electrolyte (I^−^), the dye molecule returns to its initial state (ground state) and prevents the recapture of electrons by the oxidized dye. This iodide is used to donate electrons to oxidized dyes, thus forming an electron transport cycle. With this cycle, there is a direct conversion from sunlight to electricity.

The working principle of DSSC if written in the form of an equation is as follows:

(1) $$\begin{array}{c} D+\text{hv}⟶D^{*},\\ D^{*}⟶D^{+}+{\text{e}}^{-},\\ {\text{I}}_{3}^{-}+2{\text{e}}^{-}⟶3{\text{I}}^{-},\\ 2D^{+}+3{\text{I}}^{-}⟶{\text{I}}_{3}^{-}+2D. \end{array}$$

The main issue found in DSSC is lower efficiency than silicon solar cells [10]. This low efficiency is due to obstacles in the flow of electric current in DSSC. This obstacle is caused by suboptimal contact between semiconductor particles, because there is a gap or empty space between semiconductor particles. The existence of this gap or empty space causes the current flow to be hampered; accordingly, the DSSC efficiency is low [11].

**Figure 3:**
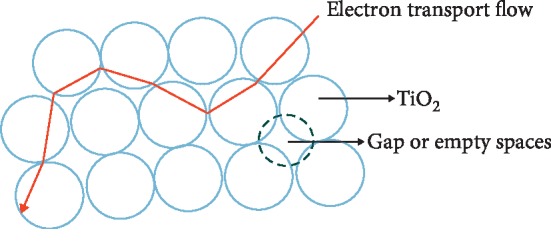
Gap or empty space between TiO_2_ [11].

In Figure 3, the flow of electron transport in the TiO2 layer, it appears that electrons are able to only flow through interconnected TiO_2_ particles because of porosity of TiO_2_ particles being able to pass through the gap or empty space [11]. This causes a decrease in DSSC efficiency.

Various attempts have been made to improve the efficiency of DSSC. Previous research has been done to improve efficiency by deposition or addition of nanosized particles to the structure of the semiconductor layer, for instance, by depositing Ag nanoparticles [12], doping Indium nanoparticles [13], adding bamboo charcoal powder particles to the TiO2 layer [14], and adding Li^+^ particles [15].

One way to increase the flow of electric current and efficiency can be done by combining organic and inorganic elements in the DSSC structure. The organic element is in the form of clathrin protein from the brain of the cow while the inorganic element is the semiconductor TiO_2_.

Clathrin is a protein found in a cow brain that plays an important role in the formation of vesicle layers. Clathrin protein in bovine brain is used to build small vesicles to transport molecules in cells. Vesicles allow cells to communicate, transfer nutrients, import signaling receptors, mediate immune responses after taking extracellular samples, and clean up cell debris left behind by tissue inflammation.

**Figure 4:**
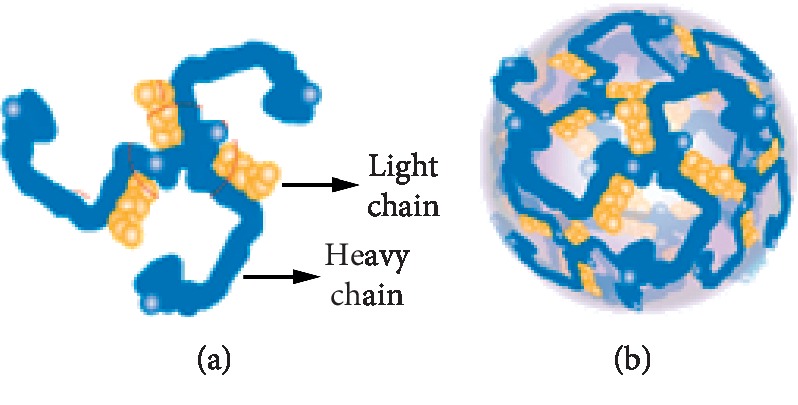
Clathrin protein [17]. (a) Single clathrin. (b) Combined clathrin.

The clathrin protein is shaped like a tripod: three leg spindles joined together on a hub or shaft. The shape of the triskelion consists of three clathrin heavy chains and three light chains. Clathrin molecules easily combine with each other to form a kind of honeycomb, similar to a lattice or net on the cell surface. When the triskelia interacts, they form a polyhedral lattice that surrounds the vesicle. Clathrin triskelion consists of three clathrin heavy chains, and each heavy chain weighing 180 kDa has a light chain of ∼30 kDa tightly bound to it. The three heavy chains provide the backbone or structural framework of the clathrin lattice, and the three light chains regulate the formation and dismantling of the clathrin lattice. When a great number of triskelia are connected, they form a basket-like structure. The clathrin combination structure is shown in Figure 4(b), built of 36 triskelia. The combination of the clathrin lattice is formed with sizes ranging 30–100 nm from about 36 clathrin molecular units consisting of 108 clathrin heavy chains and 108 clathrin light chains. When the right molecule attaches to the clathrin, the lattice structure of the clathrin will wrap it into a sac-like shape. This clathrin sac carries the molecule to its intended destination [16].

Clathrin protein derived from cow brain has been applied to various materials as a biotech substrate. Materials that have been successfully added to clathrin are graphene, polymers, glass, and metals [18]. From Schoen's research, it is known that clathrin is able to bond with titanium dioxide. Titanium dioxide is a semiconductor material in a DSSC [19]. Clathrin plays an important role in the cell transport system; to be able to transport, clathrin cells must be able to bind to ions. Examples of ions that are able to bind to clathrin are positive ions in iron [20].

This study aims to determine the effect of clathrin protein derived from cow brain in increasing efficiency of DSSC. In this research, clathrin protein from cow brain will be added to the DSSC which will cover the TiO_2_ layer and dye; therefore, it fills gaps or empty spaces in the TiO_2_ semiconductor layer resulting in better contact between TiO_2_ particles and electron transfer to a maximum because it can pass through all parts of TiO_2_ semiconductor. The addition of cow brain protein is expected to increase efficiency in DSSC.

## 2. Research Methodology

This study employed an experimental method to test the effect of the addition of cow brain protein on increased efficiency in the DSSC. The research procedure was carried out by preparing material and equipment to be used in this study, then DSSC assembly was carried out, and then testing of DSSC was made. The material preparation needed was to prepare protein from the cow brain (clathrin protein), TiO_2_ paste, dye solution, electrolyte solution, carbon counter electrode, and TCO glass. Furthermore, the following was equipment prepared: i.e., Becker glass, measuring tubes, pipettes, magnetic stirrers, petri dishes, Whatman filter paper, furnaces, digital scales, mortars, scotch tapes, multimeters, solar simulators, solar power meters, electrophoresis apparatus, and centrifuges.

Then, the assembly was carried out with the following steps: giving TiO_2_ paste to the electrode glass, heating glass/TiO_2_, soaking the dye, giving cow brain protein, giving electrolytes, giving carbon to the electrode counter, and then DSSC assembling. After the DSSC was assembled, tests which include XRD testing, FTIR testing, SEM testing, and efficiency testing in the solar simulator were performed.

Clathrin protein was obtained from protein isolation of a cow brain carried out in Biochemistry Laboratory of Universitas Brawijaya. The steps of protein isolation and making a protein profile with SDS-PAGE from cow brain tissue were as follows: doing homogenization of 300 mg of cow brain samples and then sonification for about 10 minutes. The next step was centrifuging on 6000 rpm for 15 minutes to get supernatant layer on top and its sediment at the bottom layer. After separating the supernatant from sediment, then supernatant was added with ethanol in a volume ratio of 1 : 1. Ethanol was added to the uppermost layer containing plasmids and left to form sediment at −20°C. The next step was to recentrifuging at 10000 rpm and obtain pellets in very small amounts. Then, Tris-HCl solvent was added with a volume ratio of 1 : 1 which serves to maintain the pH of the solution. The next step was to do protein profiles with Sodium Dodecyl Sulfate-Polyacrylamide Gel Electrophoresis (SDS-PAGE) technique which is a small-scale purification technique that results in the separation of a protein based on its molecular weight in specific bands that appear on polyacrylamide gels. After the protein profile process, then sample was immersed in a distaining solution while shaking using a shaker until the bands on the gel is clearly visible. The bands in the electrophoresis gel were determined by their respective Retention factor value (Rf value) and relative molecular masses and then recorded in the form of data.

TiO_2_ paste was made as follows: Polyvinyl Alcohol (PVA) of 1.5 grams was added to 13.5 ml of distilled water, and then it stirred with a rotary motor at a temperature of 80°C for ± 30 minutes until the solution thickens. This suspension functioned as a binder in making paste. Add the suspension to 0.5 grams of TiO_2_ powder which is about 7.5 ml. TiO_2_ powder used is titanium dioxide anatase from Sigma-Aldrich. Then, a mixture of suspension and TiO_2_ powder was crushed by a mortar to form a good paste to be coated. The optimal degree of viscosity of the paste was obtained by adjusting the number of binders and also if necessary water was added to the mixture of binder and TiO_2_ powder.

Electrolyte was prepared as follows: electrolyte solution was made by dissolving a mixture of 0.8 grams (0.5 M) of potassium iodide (KI) into 10 ml of PEG 400 and then stirring evenly. Furthermore, 0.127 grams (0.05 M) of iodine (I_2_) was added to the solution and stirred until all three ingredients were completely dissolved. The ready-to-use electrolyte solution was temporarily stored in an enclosed dark bottle.

The dye was prepared as follows: 100 grams of papaya leaves with acetone ProAnalysis (P.A) 500 ml (w/v) solvent was prepared for extraction. The papaya leaves were firstly cleaned and drained. Next, the leaves were cut into small pieces and blended until they were smooth. The finer the papaya leaves, the better the extraction process. Papaya leaves that have been refined were then mixed with 500 ml acetone P.A into a Becker glass. Furthermore, it was stirred using a rotary motor for 30 minutes; thus, the chlorophyll was separated from the leaves, until the acetone P.A solution turned into green and the leaves turned into white. Then, the contents of the glass were filtered using gauze paper (Whatman) to separate the solution with leaf pulp. Consequently, we obtained a 500 ml dye from the papaya leaf. After that, the dye was kept in a tightly closed dark bottle to avoid decomposition and light which reduce the absorption of the dye.

Hereinafter, carbon counter electrodes were composed as follows: one of the TCO glass substrates which acts as a counter electrode was burned using a candle flame until the soot filled the conductive area of the substrate. This combustion process coated the substrate with carbon (carbon counter electrodes). This carbon served as a catalyst in DSSC. The catalyst was needed to accelerate the reaction kinetics of the triiodide reduction process on TCO.

The following procedure explains DSSC assembly: on the conductive TCO glass that has been cut into 1.5 × 1.5 cm^2^ where TiO_2_ was deposited by scotch tape on the side of the glass that has resistivity, thus, it formed 1 × 1 cm area. Scotch tape was also used to control the thickness of TiO_2_ paste. To thicken the paste on the glass surface, scotch tape can be stacked in layers according to the needs.

TiO_2_ paste was deposited over the area that has been made on the conductive glass by the doctor blade method, with the aid of a stirring rod to flatten the TiO_2_ paste on the substrate starting from the end of the frame. Then, the conductive transparent glass coated with TiO_2_ paste was dried in a furnace at 450°C for 30 minutes. This process aims to grow porosity and form a good adhesive contact between the solution and the TCO glass substrate.

Conductive glass has been deposited with TiO_2_ paste and has been heated and then immersed in a dye solution for approximately 24 hours, and then the TiO_2_ layer on the conductive glass turned into green. In this process, chlorophyll adsorption occurred on the surface of TiO_2_.

Furthermore, cow brain protein solution was added to the layer of TiO_2_ and dye with varying concentrations of 25%, 50%, and 75% to TiO_2_. Then, the electrolyte solution was dripped on a layer of TiO_2_/dye/cow brain protein.

The final step in manufacturing DSSC was to unite the two substrates. The counter electrode which has been given a carbon catalyst was then placed on a layer of TiO_2_/dye/cow brain protein/electrolyte with a sandwich structure where each tip was offset by 0.5 cm for electrical contact. Then, the cell structure was firmly clamped with binder clips on both sides. DSSC solar cells were ready to be tested.

Tests carried out on DSSC were XRD testing to determine the crystal structure and crystal size, FTIR testing to determine the functional groups formed on DSSC, SEM testing to determine the surface morphological characteristics of the DSSC layer, and testing electric current and voltage to determine the changes in efficiency that occurred in DSSC.

Electric current and voltage on DSSC were generated by lighting using halogen lamps in a solar simulator, and then the change in current and voltage produced was measured using a data logger to determine DSSC performance. Schematically, the research installation is as follows.

**Figure 5:**
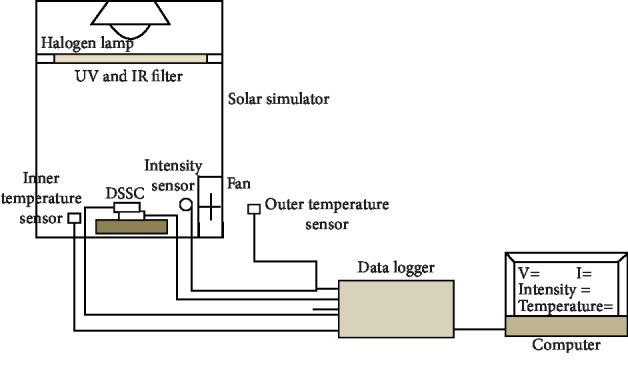
Research instrument installation.

It consisted of a solar simulator to provide lighting to DSSC to produce current and voltage to be measured to determine DSSC performance as shown in Figure 5. The solar simulator has a length of 300 mm, width of 300 mm, and height of 500 mm. The equipment in the solar simulator is a 1000 W/m^2^ intensity halogen lamp to provide lighting, UV and IR filters to produce visible light, temperature sensors, intensity sensors, and fans that function to maintain a constant temperature. Current and voltage data generated in DSSC were measured using the Arduino Uno ATmega 328 data logger microcontroller.

The stages of data collection are as follows: the equipment was set according to Figure 5. Then, DSSC was placed in the middle with the distance to the lighting regulated; thus, the intensity was 1000 W/m^2^. Then, the lights were turned on; accordingly, current and voltage emerged from DSSC. DSSC varied the percentage of clathrin protein content in TiO_2_, namely, 0%, 25%, 50%, and 75%. Current and voltage data obtained were then transferred to a computer to calculate its efficiency (it followed the method suggested by Trihutomo et al.) [21].

## 3. Results and Discussion

### 3.1. XRD Testing

**Figure 6:**
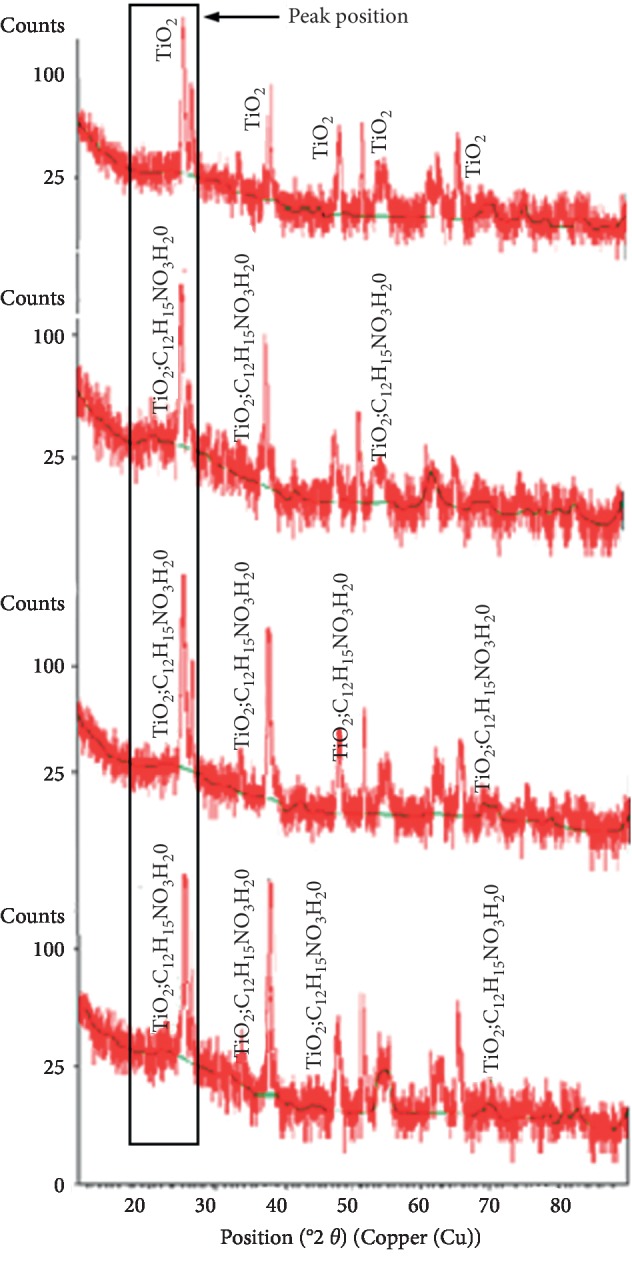
Diffractogram XRD test results for each cow brain protein concentration (%) (a) 0, (b) 25, (c) 50, and (d) 75.

X-Ray Diffractometer (XRD) testing was used to determine the crystal structure formed and grain size in the TiO_2_ layer doped with cow brain protein. The XRD test results in the form of a diffraction pattern (diffractogram) consisting of characteristic peaks for each concentration of protein addition on TiO_2_ are presented in Figure 6.

Based on Figure 6, it appears that the peaks formed from the crystal structure are sharp because they have a high degree of orderliness, whereas in amorphous, the peaks generated were very gentle slopes since they have a very low degree of order.

In Figure 6(a), based on reference code 01-073-1764 from the International Center for Diffraction Database (ICDD) data, the crystal structure formed was TiO_2_ type anatase. The use of anatase phase TiO_2_ nanoparticles in DSSC has the potential to achieve higher efficiency in converting light into electricity because it has a high photoactive ability. In Figures 6(b)–6(d), with the addition of protein to TiO_2_, based on reference code 00-049-2369, the crystalline structure of amino acid hydrate (C12H15NO3H20) appears in intensity which increases with increasing concentration of cow brain protein. The appearance of the amino acid crystal structure indicates the successful synthesis of cow brain protein in TiO_2_. The presence of amino acids in the DSSC structure increases the conductance of electric current in the DSSC layer since amino acids are electrolyte. This is because amino acids have a structure that tends to be charged and has a high polarity. Increasing the electric current will increase efficiency on DSSC. Amino acids have the ability as zwitterions, a molecule that contains both a carboxylic group (acidic) and an amine group (basic). By having the zwitterions capability, amino acids simultaneously contain negative ions and positive ions in the molecule. Electrons will be captured by amino acid molecules when an acid reaction occurs and electrons will be released by amino acid molecules when a base reaction occurs [22]. Efficiency increased in DSSC because amino acids in cow brain proteins can act as electron bridges between porous TiO_2_ molecules so that electron transfer is faster and reduces recombination due to the effects of zwitterions contained in cow brain proteins.

The XRD test results shown in Figures 6(a)–6(d) in the peak position area (marked with a square) show the intensity of the diffraction peak that are getting higher and firmer with the addition of 25%, 50%, and 75% protein concentrations in TiO_2_. With the higher intensity due to the addition of clathrin protein, the degree of crystallinity of the sample is better. With a good degree of crystallinity, the electron diffusion process in TiO_2_ will be faster which implies that the electron transfer process for DSSC as a whole will be higher; hence, it will increase the efficiency of solar cells. It is clear from Figures 6(b)–6(d) in the peak position area that after adding clathrin protein the count or intensity of diffractogram pattern is higher than Figure 6(a) without clathrin protein; this indicates the growth of crystallites due to the addition of clathrin protein on TiO_2_.

The growth of crystallites causes a better degree of crystallinity so that the electron transfer is better and increases the efficiency of DSSC [23]. The increase in intensity due to the addition of clathrin can also be proven from the Full Width at Half Maximum (FWHM) value. Based on Table 2, with increasing clathrin concentration, it appears that the FWHM value is getting smaller. The smaller FWHM value associated with a sharper diffraction peak or higher diffraction intensity (marked with a square) indicates the growth of crystals so that the degree of crystallinity is getting better [24].

The diffractogram pattern obtained from XRD data can also be used to determine the size of the crystals formed based on the FWHM values at various peak position using the Scherrer equation, *D*=(*k* · *λ*)/*β*cos*θ* , where *D* is the size of the crystal, *λ* = 0.154 nm is the wavelength of X-rays, *β* is the FWHM value of each characteristic peak position, *θ* is the diffraction angle of peak position, and *k* ≈ 0.94 is a constant.

The peak position of the diffractogram pattern is determined in the area marked by the squares (Figure 6) because it has the highest intensity which indicates the best degree of crystallinity. The *x*-axis on the diffractogram states the diffraction angle position (°2*θ*) which functions to determine the phase of the material formed. The *y*-axis on the diffractogram states the amount of intensity from the diffraction angle of the material phase; the higher intensity indicates better degree of crystallinity of the material.

From Table 2, it is known that at peak position (°2*θ*) for each concentration of clathrin protein addition the FWHM value decreases with increasing clathrin concentration. The smaller FWHM value is related to the sharper diffraction peaks produced. The sharper diffraction peaks produced are related to the smaller FWHM. As it is known that the smaller FWHM indicates an increase in crystallinity characterized by crystallite growth [24], the growth of this crystallite can be represented by the size of the crystallite produced in each sample. Thus, the crystallinity of titania with the addition of clathrin in this sample group (25%, 50%, and 75%) increases with increasing clathrin concentration. This analysis is also strengthened by the results of large crystallite calculations using the Scherrer equation. The estimated results of the sample size calculation of crystallites are 0%, 25%, 50%, and 75% based on the Scherrer equation as explained in Table 2.

From Table 2, it appears that at peak position (°2*θ*) for each concentration the addition of clathrin protein is known with the increasing percentage of cow brain protein in TiO_2_ leading to the increase of crystal size. The greater size of the crystals indicates the incorporation of cow brain protein molecules in the TiO_2_ molecule. The larger crystal size affects the distance of the atoms in the crystal that are increasingly close altogether resulting in a smaller lattice strain between molecules; accordingly, the internal resistance to the flow of electrons between molecules is also reduced [25].

### 3.2. FTIR Testing

**Figure 7:**
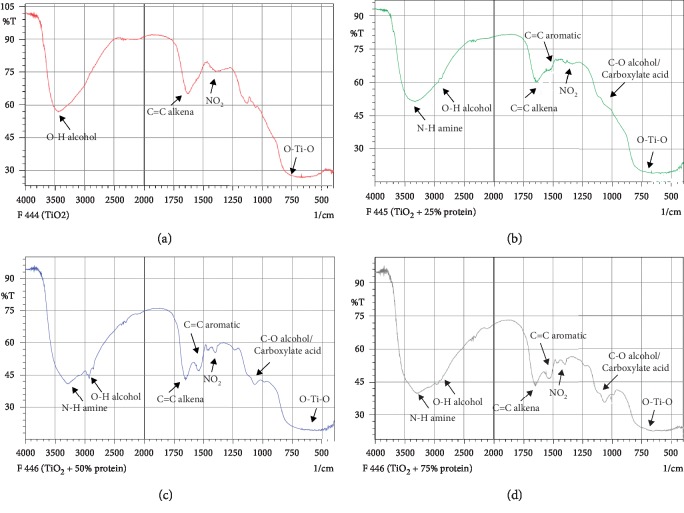
FTIR testing results on each cow brain protein concentration (%). (a) 0, (b) 25, (c) 50, and (d) 75.

Fourier Transform Infrared Spectroscopy (FTIR) testing was used to determine the functional groups formed in the DSSC layer due to the addition of cow brain protein. FTIR test results for each concentration of cow brain addition on TiO_2_ can be seen in Figure 7.

From the results of the FTIR testing shown in Figure 7, it appears that the greater concentration of cow brain protein on DSSC shows the steeper absorption at the transmittance rate of the wave numbers in the FTIR spectrum. With the steeper uptake, the greater energy was absorbed by the functional group formed by the increasing concentration of cow brain protein in DSSC.

Based on the results of FTIR testing that have been carried out, it can be seen that the functional groups were formed. The results of FTIR testing showed peaks appearing in the wave number 500–900 cm^−1^ indicating the presence of TiO_2_ groups at all clathrin protein concentrations, peaks appearing in wave numbers of 1050–1150 cm^−1^ in samples of TiO_2_ + 50% cow brain protein, TiO_2_ + 75% cow brain protein showing the presence of CO Alcohol/Carboxylic Acid groups, peaks appearing in the wave number 1500–1570 cm^−1^ in samples of TiO_2_ + 25% cow brain protein, TiO_2_ + 50% cow brain protein, TiO_2_ + 75% cow brain protein showing the NO2 group of Nitro compounds, peak appearing at wave numbers 1500–1600 cm^−1^ at samples of TiO_2_ + 25% cow brain protein, TiO_2_ + 50% cow brain protein, and TiO_2_ + 75% cow brain protein showing the presence of C=C Aromatic Ring groups, peaks appearing in the wave number 1610–1680 cm^−1^ indicating the presence of C= groups C. Alkene, peaks appearing at wave numbers 2500–3600 cm^−1^ indicating the presence of OH groups Hydrogen bonding, and peaks appearing at wave numbers 3300–3500 cm^−1^ indicating the presence of NH Amine/Amide groups [26]. The formation of functional groups in DSSC are N-H compounds amine, O-H, alkene, aromatic, NO-nitro compounds, and C-O alcohol/carboxylic acid that show functional groups of elements of amino acids so that they also form proteins [27].

From the results of the FTIR testing in Figures 7(b)–7(d), it appears that the higher the concentration of clathrin on TiO_2_, the sharper the uptake that occurred in the FTIR spectrum wave numbers for the protein constituent groups. The sharper uptake intensity indicates the higher structure or functional groups of amino acids formed and indicates the greater energy absorbed by the functional groups formed from the greater increase in the concentration of clathrin protein in DSSC [28].

Amino acids which simultaneously contain amine groups and carboxylic groups can be seen to have the ability as zwitterion. Zwitterion has the ability of molecules that have simultaneously negative ions and positive ions in the molecule. Zwitterion has the ability to do both acid and base reactions in its molecules [22]. When an acid reaction occurs, the molecule will capture electrons and when a base reaction occurs the molecule will release electrons. The zwitterion effect of the clathrin protein from cow brains provides the ability to transfer electrons and increase efficiency in DSSC.

### 3.3. SEM Testing

**Figure 8:**
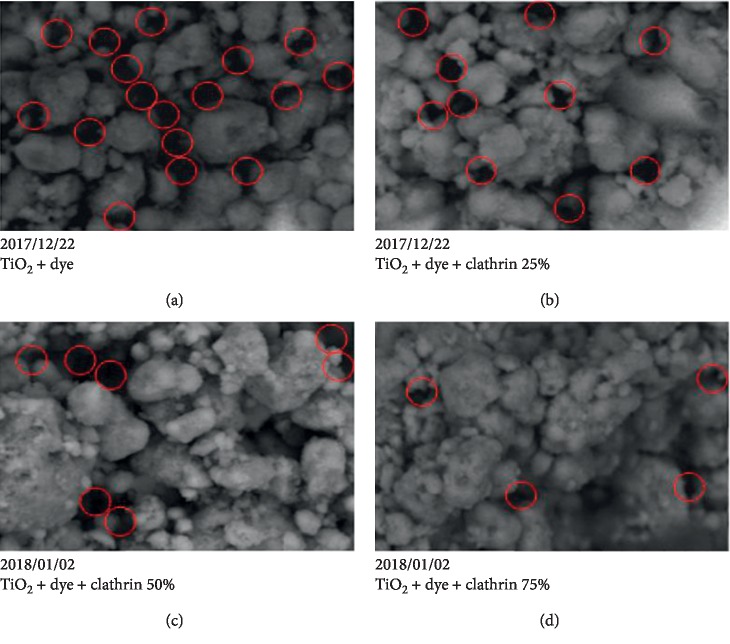
DSSC morphology of 5000x magnification for each clathrin percentage (%) (a) 0, (b) 25, (c) 50, and (d) 75.

SEM (Scanning Electron Microscopy) analysis aims to determine the surface morphological characteristics of the DSSC layer. Figures 8(a)–8(d) show a DSSC morphology with a variation of the percentage of clathrin to TiO2, i.e., 0%, 25%, 50%, and 75% SEM analysis results with magnification of 5000x.

Figure 8 shows SEM image with a magnification scale of 5000x of TiO2 nanomaterials that has been coated on FTO glass; it appears that the surface of this thin layer is not flat with the presence of light and dark parts, where the light part is coated with TiO_2_/clathrin/dye layers while the dark parts are a cavity or gap that is not covered by a layer and are between the TiO_2_/clathrin/dye layers.

Figure 8(a) shows that the surface of the TiO2 thin film is hollow or porous. In the figure, it appears that there are still many cavities between TiO_2_ molecules that are marked with a lot of dark areas. Cavities in this thin layer function for adsorption of dye molecules in TiO_2_ [29]. But the presence of these cavities causes the transfer of electrons between TiO_2_ molecules to be inhibited, and this causes DSSC performance to be low. From the SEM test results, Figures 8(b)–8(d) show that the concentration of additional molecules of protein deposited into TiO_2_ increases, and the cavity or pore between the TiO_2_ molecules decreases; this can be seen by the decreasing portion of the dark area. The reduction of cavities in the layers indicates that protein molecules fill cavities that exist between TiO_2_ molecules.

The addition of clathrin that fills the gap between TiO_2_ particles can improve connectivity between TiO_2_ particles and produce closer contact of the semiconductor oxide, thereby accelerating the transfer of charge in the photoanode. Increased connectivity between TiO_2_ particles causes a reduction in electron flow resistance and creates a short path of electron flow, thereby reducing the possibility of recombination, and can increase DSSC efficiency. The presence of deposited molecules derived from these proteins serves as a bridge of electron transport to the anode becoming faster because proteins are electrolyte. The electrolytic character of proteins is due to the constitution making up of proteins, because protein molecules contain over 200 amino acid residues or more condensed into long polypeptide chains. Protein as an electrolyte is amphoteric that can act as a cation or anion. The zwitterions effect of proteins is that proteins can carry both positive and negative charges at the same time. Cations can join proteins if anionic-charged proteins are in an alkaline state while anions can join proteins if cationic-charged proteins are in an acidic state [30]. The presence of this electron bridge causes many interconnected molecules to facilitate faster electron transport so as to reduce recombination and improve DSSC performance.

### 3.4. Efficiency Testing

Testing of electrical current and voltage aims to determine changes in efficiency that occur after an increase in the concentration of cow brain protein by 0%, 25%, 50%, and 75% on DSSC. Testing of electric current, voltage, and efficiency on DSSC with the addition of cow brain protein of 0%, 25%, 50%, and 75% was done by measuring changes in electric current and voltage that occur under a beam of 1000 W/m^2^ halogen lamps. The results of testing electric current, voltage, and efficiency for each additional concentration of bovine brain protein are presented in Table 3.

From Table 3, it can be seen that the increase in the concentration of cow brain protein added to DSSC causes an increase in the resulting efficiency. The highest efficiency value on DSSC was the addition of 75% protein concentration with 1.465% efficiency value.

The increase in the value of efficiency in DSSC is due to the cow brain protein molecule added to DSSC which fills the gaps or empty spaces in TiO_2_ because the clathrin protein derived from cow brain is zwitterions that is able to act as an electron bridge between porous TiO_2_ particles so as to reduce obstacles from the flow of electric current resulting in increased efficiency [31]. Electron bridges are able to occur because of the ability of proteins to do both acid and base reactions at once.

As one type of protein, clathrin is composed of various amino acids. According to Lewis's theory, amino acids are able to conduct electrons [32]. Amino acids are compounds making up proteins. Amino acids have one carboxyl group and one amino group. In general, the amino group is bound to the position of the carboxyl group. Amino acids are able to act as acids (donating protons to strong bases) and to act as bases (receiving protons from strong acids).

According to the Lewis acid base theory, what is meant by Lewis acid is a compound that is able to accept electron pairs from other compounds, or electron pair acceptors, while Lewis bases are compounds that are able to render electron pairs to other compounds or donor electron pairs.

Lewis Acid Base Theory example is as follows.

**Figure 9:**
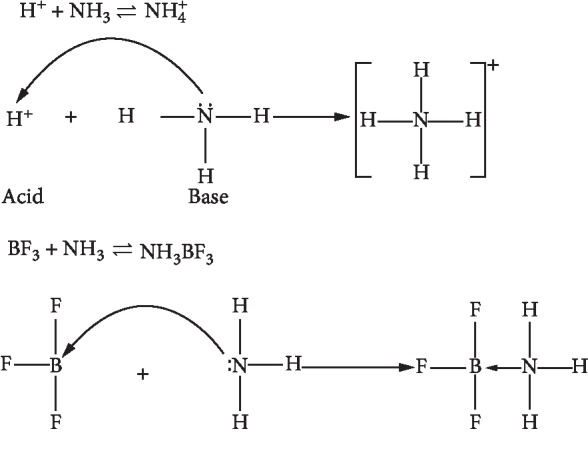
Lewis acid base theory [32].

In Figure 9, it is shown that, in the H^+^ and NH_3_ reactions, the H^+^ ion is a Lewis acid because it is able to accept electron pairs, whereas NH_3_ is a Lewis base. In the reaction between BF_3_ and NH_3_, BF_3_ is Lewis acid because it is able to accept a pair of electrons, while NH_3_ is a Lewis base.

**Figure 10:**
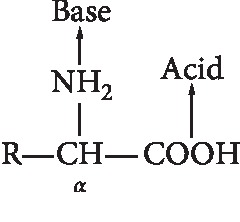
Amino acids structure [33].

Amino acids are compounds that consist of one or more carboxyl groups (−COOH) and one or more amino groups (−NH_2_), one of which is located on the C atom right next to the carboxyl group (C alpha atom). Amino acids combine through peptide bonds, which are bonds between carboxyl groups of amino acids and amino groups of amino acids that are next to it, as shown in Figure 10 [33].

**Figure 11:**
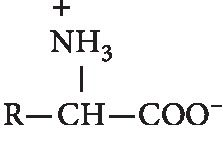
Zwitterion structure of amino acids [34].

The presence of unrestrained amino and carboxyl groups at the ends of a chain of protein molecules causes proteins to have a lot of charge and be amphoteric (able to react with acids or bases). In an acid solution, the amino group reacts with H^+^; accordingly, the protein is positively charged. When this condition is carried out, electrolysis, protein molecules will move towards the cathode. And conversely, in alkaline solutions protein molecules will react as acids or negatively charged; hence, protein molecules will move towards the anode as shown in Figure 11 [34].

The functional groups structurizing amino acids are able to enhance the electron transfer process in the DSSC layer since amino acids are amphoteric that are able to act as acids (donating protons to strong bases) and act as bases (receiving protons from strong acids) [22]. Amino acids are compounds making up proteins. Amino acids have one carboxyl group and one amino group.

Amino acids in the form of zwitterion, i.e., carboxyl groups in amino acids, are able to release hydrogen ions to be negatively charged and amine groups are able to receive hydrogen ions to be positively charged.

**Figure 12:**
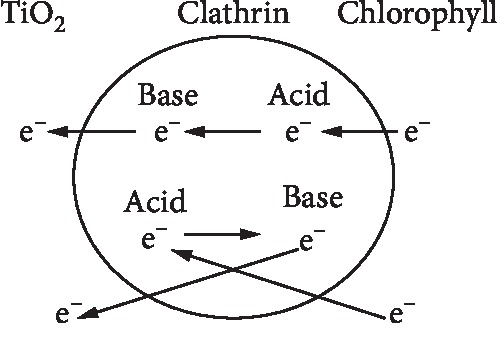
Zwitterion phenomenon on gap between TiO_2_.

Figure 12 explains the phenomenon that occurs in the gap or empty space between TiO2 molecules that have been filled with clathrin protein; that is, when electrons flow from chlorophyll, they will be captured by clathrin protein molecules through an acidic reaction, and in this acidic state, the protein will donate protons to the base; hence, the protein tends to contain electrons and is eligible to be considered as negative poles (anodes), whereas in base reactions, proteins will receive protons from acids so proteins tend to contain protons and are eligible to be considered as positive poles (cathodes). In this state, electrons will flow from acids to bases. Through an alkaline reaction, the electrons will be released into TiO2; hence, the electron flow from chlorophyll to TiO_2_ will pass through the clathrin protein electron bridge. Accordingly, the electrons are able to go through all the pathways between TiO_2_ particles and increase the electric current in DSSC.

**Figure 13:**
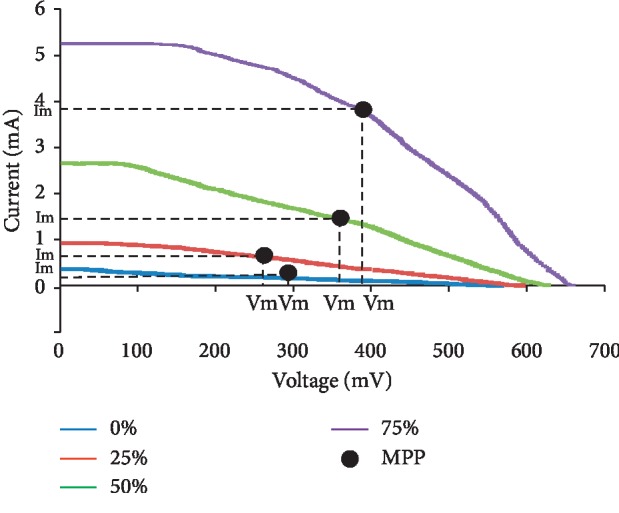
Comparison of electric current and voltage at each protein concentration.

Comparison of the characteristics of DSSC test results of electric current and voltage for each concentration of cow brain protein addition is presented in Figure 13.

From Figure 13, it appears that the addition of clathrin concentration causes the short circuit current (Isc) and the open circuit voltage (Voc) to increase. This increase in current and voltage will result in an increase in DSSC efficiency. Yet, the movement of electrons in the TiO_2_ layer towards the electrodes causes a decrease in current due to the higher resistance caused by the distance travelled by electrons also getting farther away in the TiO_2_ layer which is getting thicker.

**Figure 14:**
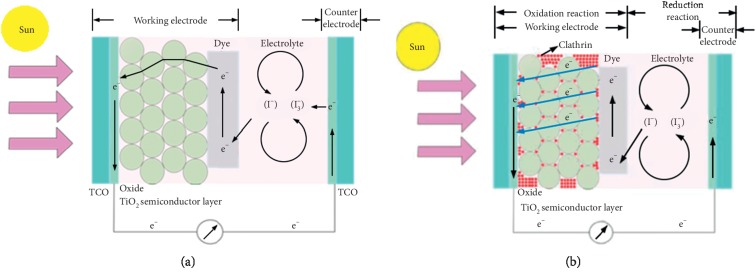
Electrons flows mechanism on DSSC. (a) DSSC without clathrin. (b) DSSC with clathrin.

Figure 14 illustrates the mechanism of DSSC without clathrin and DSSC with clathrin. In the DSSC mechanism without clathrin, it appears that electrons flowing in the TiO_2_ conduction area are able to only flow through interconnected TiO_2_ particles; hence, the electron pathway is longer and the possibility of recombining electrons with oxidized dyes is greater, whereas in the DSSC mechanism with the addition of clathrin, it appears that the clathrin added fills the gap or empty space between the TiO_2_ particles; accordingly, the electron path in the TiO_2_ conduction area is shorter and the electrons are able to pass through all parts of the TiO_2_ particle and cause internal resistance to electron flow to decrease and the possibility of electron recombination with dye oxidation decreases which results in an increase in DSSC efficiency.

**Figure 15:**
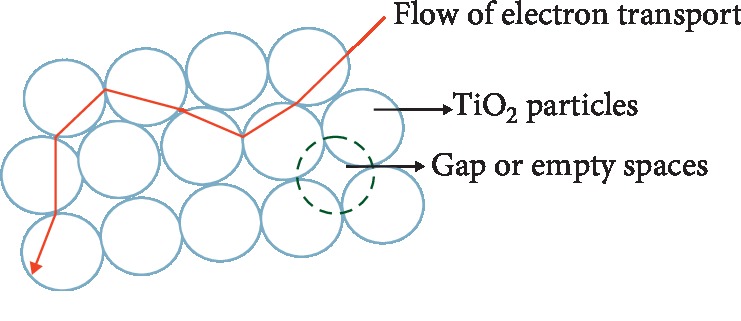
Internal resistance on conventional DSSC [11].

In conventional DSSC, there are gaps or empty spaces at the grain boundary of the combined TiO_2_ molecules. These gaps or empty spaces cause obstacles from electron transport injected into the TiO_2_ semiconductor. Due to the resistance of the internal resistance to TiO2, this electron diffusion is inhibited, resulting in a higher chance of recombining electrons to dye and electrolytes. It is illustrated in Figure 15.

**Figure 16:**
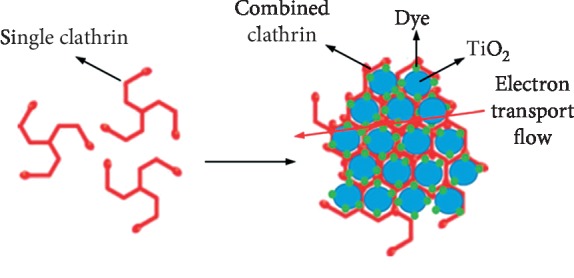
Electron flows and DSSC molecules structure of DSSC added with clathrin.

In the DSSC added with clathrin, clathrin molecules have the ability to self-assemble and to wrap particles, and the combining clathrin molecules around 30–100 nm in size will wrap around TiO_2_ about 11–20 nm in size and fill gaps or empty spaces between grain boundaries of TiO_2_. Accordingly, it reduces internal resistance and generates maximum electron transport. The flow of electrons and the molecular structure of the layer after the addition of clathrin is illustrated in Figure 16.

## 4. Conclusion

According to the results and discussion explained above, this research presents several conclusions as follows:

1. The addition of cow brain protein forms amino acid constituent functional groups in DSSC in the form of the emergence of N-H compounds Amine, O-H, Alkene, Aromatic, NO2 nitro compounds, and C-O Alcohol/Acid, while amino acids are compounds making up proteins.
2. The more concentration of cow brain protein on DSSC indicates the steeper absorption at the transmittance rate of the wave numbers in the FTIR spectrum. The steeper uptake indicates the greater energy absorbed by the functional group formed by the increasing concentration of cow brain protein in DSSC.
3. When the number of concentrations of additional molecules of proteins deposited into TiO_2_ is increasing , the cavity or pore between the existing TiO_2_ molecules decreases. The reduction of cavities in the layers indicates that protein molecules fill cavities that exist between TiO_2_ molecules. The addition of clathrin that fills the gap between TiO_2_ particles can improve connectivity between TiO_2_ particles and produce closer contact of the semiconductor oxide, thereby accelerating the transfer of charge in the photoanode.
4. An increase in the concentration of cow brain protein added increases DSSC efficiency. The highest efficiency value on DSSC is the addition of 75% protein concentration with 1.465% efficiency value.
5. The efficiency value increase on DSSC is due to the cow brain protein molecule added to DSSC which fills a gap or empty space in TiO_2_. Cow brain protein contains zwitterion properties that is able to act as electron bridges between porous TiO_2_ particles; accordingly, it is capable of reducing the resistance of the flow of electric current and increasing efficiency produced.

## Figures and Tables

**Table 1:** Comparison of natural dye efficiency on DSSC [4].

Dye	Jsc (mAcm^2^)	Voc (V)	FF	*η* (%)
Rosella	1.63	0.4	0.57	0.37
Blue pea	0.37	0.37	0.33	0.05
Mixed rosella-blue pea	0.82	0.38	0.47	0.15
Black rice	1.14	0.55	0.52	
Capsicurn	0.23	0.41	0.63	
Rosa xanthina	0.64	0.49	0.52	
Kelp	0.43	0.44	0.62	
*Erythrina variegata*	0.78	0.48	0.55	
Bixin	1.1	0.57	0.59	0.37
Annatto	0.53	0.56	0.66	0.19
Norbixin	0.38	0.53	0.64	0.13
Crocetin	2.84	0.43	0.46	0.56
Crocin	0.45	0.58	0.6	0.16
Fruit of calafate	6.2	0.47	0.36	
Syrup of calafate	1.5	0.38	0.2	
Skin of jaboticaba	7.2	0.59	0.54	
Red Sicilian orange	3.84	0.34	0.5	
Purple eggplant extract	3.4	0.35	0.4	
Dragon fruit	0.2	0.22	0.3	0.22
Pomegranate juice	0.2	0.4	0.45	1.5
Red turnip	9.5	0.43	0.37	1.7
Wild Sicilian prickly pear	8.2	0.38	0.38	1.19
Sicilian Indian fig	2.7	0.38	0.54	0.5
*Bougainvillea*	2.1	0.3	0.57	0.36
Shisonin	3.56	0.55	0.51	1.01
Shisonin and chlorophyll	4.8	0.53	0.51	1.31
Chlorophyll	3.52	0.43	0.39	0.59
*Hibiscus surattensis*	5.45	0.39	0.54	1.14
*Sesbania grandiflora*	4.4	0.41	0.57	1.02
*Hibiscus rosa-sinensis*	4.04	0.4	0.63	1.02
*Nerium oleander*	2.46	0.41	0.59	0.59
*Ixora macrothyrsa*	1.31	0.4	0.57	0.3
*Rhododendron arboreum* zeylanium	1.15	0.4	0.64	0.29
*Begonia*	0.63	0.54	0.72	0.24
Tangerine peel	0.74	0.59	0.63	0.28
*Rhododendron*	1.61	0.59	0.61	0.57
*Fructus lycii*	0.53	0.69	0.47	0.17
Marigold	0.51	0.54	0.83	0.23
*Perilla*	1.36	0.52	0.7	0.5
Herba artemisiae scopariae	1.03	0.48	0.68	0.34
China loropetal	0.84	0.52	0.63	0.27
Yellow rose	0.74	0.61	0.57	0.26
Flowery knotweed	0.6	0.55	0.63	0.21
Bauhinia tree	0.96	0.57	0.66	0.36
Petunia	0.85	0.62	0.61	0.32
*Lithospermum*	0.14	0.34	0.59	0.03
Violet	1.02	0.5	0.65	0.33
Chinese rose	0.9	0.48	0.62	0.27
Mangosteen pericarp	2.69	0.67	0.63	1.17
Rose	0.97	0.6	0.66	0.38
Lily	0.51	0.5	0.67	0.17
Coffee	0.85	0.56	0.69	0.33
Broadleaf holly leaf base	1.19	0.61	0.65	0.47
Red *Bougainvillea glabra*	2.34	0.26	0.74	0.45
Violet *Bougainvillea glabra*	1.86	0.23	0.71	0.31
Red *Bougainvillea spectabilis*	2.29	0.28	0.76	0.48
Violet *Bougainvillea spectabilis*	1.88	0.25	0.73	0.35
Spinach	0.47	0.55	0.51	0.13
*Ipomea*	0.91	0.54	0.56	0.28
*Bougainvillea brasiliensis*	5	0.25	0.36	0.45
*Garcinia subelliptica*	6.48	0.32	0.33	0.69
*Ficus spathacea*	7.85	0.52	0.29	1.18
Rhoeo spathacea	10.9	0.5	0.27	1.49

**Table 2:** Crystal Structure Peak and Crystal Size Data for each concentration of protein clathrin addition on TiO_2_.

Clathrin (%)	Peak position (°2*θ*)	FWHM (radian)	Crystal size (nm)
0	25.22	0.0062	24.17
25	25.18	0.0055	26.35
50	25.31	0.0048	30.15
75	25.09	0.0034	42.25

**Table 3:** Electric current, voltage, and efficiency at each concentration of cow brain protein testing results.

DSSC (%)	Isc (mA)	Voc (mV)	Im (mA)	Vm (mV)	FF	*η* (%)
0	0.353	562	0.161	293	0.238	0.047
25	0.917	590	0.608	269	0.302	0.164
50	2.643	624	1.368	377	0.313	0.516
75	5.247	657	3.836	382	0.425	1.465

## Data Availability

The data used to support the findings of this study are available from the corresponding author upon request.
